# How to govern the digital transformation of health services

**DOI:** 10.1093/eurpub/ckz165

**Published:** 2019-11-18

**Authors:** Walter Ricciardi, Pedro Pita Barros, Aleš Bourek, Werner Brouwer, Tim Kelsey, Lasse Lehtonen, Expert Panel on Effective Ways of Investing in Health (EXPH), Christian Anastasy, Pedro Barros, Margaret Barry, Aleš Bourek, Werner Brouwer, Jan De Maeseneer, Dionne Kringos, Lasse Lehtonen, Martin McKee, Liubove Murauskiene, Sabina Nuti, Walter Ricciardi, Luigi Siciliani, Claudia Wild

**Affiliations:** 1 Hygiene section, Public Health Institute, Università Cattolica del Sacro Cuore, Rome, Italy; 2 Department of Woman and Child Health and Public Health - Public Health Area, Fondazione Policlinico Universitario A. Gemelli IRCCS, Rome, Italy; 3 Nova School of Business and Economics, Universidade Nova de Lisboa, Portugal; 4 Masaryk University, Faculty of Medicine, Center for Healthcare Quality, Brno, Czech Republic; 5 Erasmus School of Health Policy & Management, Erasmus University Rotterdam, The Netherlands; 6 Australian Digital Health Agency, Sidney, Australia; 7 Department of Public Health, Helsinki University Hospital and University of Helsinki, Helsinki, Finland

## Abstract

The impact of digitalization of health services has been profound and is expected to be even more profound in the future. It is important to evaluate whether digital health services contribute to health system goals in an optimal way. This should be done at the level of the service, not the ‘digital transformation’. Decisions to adopt new digital health services, at different levels of the health care system, are ideally based on evidence regarding their performance in light of health system goals.

In order to evaluate this, a broad perspective should be taken in evaluations of digital health services. Attainment of the broad health system goals, including quality, efficiency and equity, are objectives against which to judge new digital health services. These goals in a broad sense are unaltered by the process of digitalization. Governance should be designed and tailored in such a way to capture all relevant changes in an adequate way.

When evaluating digital health services many specific aspects need to be considered.

Like for other innovations and (new) technologies, such promises may or may not materialize and potential benefits may also be accompanied by unintended and/or negative (side) effects in the short or long term. Hence, the introduction, implementation, use and funding of digital health technologies should be carefully evaluated and monitored.

Governments should play a more active role in the further optimization both of the process of decision making (both at the central and decentral level) and the related outcomes. They need to find a balance between centralized and decentralized activity. Moreover, the broader preparation of the health care system to be able to deal with digitalization, from education, through financial and regulatory preconditions, to implementation of monitoring systems to monitor its effects on health system performance remains important.

## Digitalization and health

Health technologies, in the widest meaning of the word, have changed continuously ever since the early stages of medicine. Increasing knowledge and diagnostic, preventive, treatment and rehabilitation possibilities have altered the content of health care systems. In turn, health systems have also evolved into complex entities with changing roles and responsibilities for patients, health professionals, payers and regulators. The ‘digital transformation of health services’ is seen as an important and influential process, that has already had a substantial impact on current health care and health systems and is expected to have a further fundamental impact on health care and health care delivery in the future.

**Figure 1:**
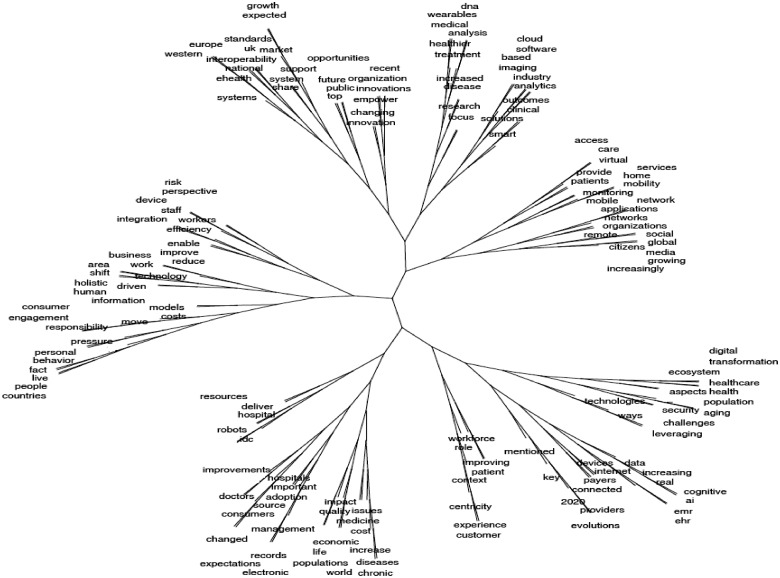
Illustrating the complexity of the digital transformation of health services. *Source:* Expert Panel on Investing in Health, European Commission, 2018

It is also immediately acknowledged that ‘the digital transformation of health services’ is a complex and multifaceted issue. The scale of impact, areas affected and complexity of the interactions of the digital with health service provision are illustrated in the topic tree shown in figure 1.^1^ This topic tree was based on clustering concepts in a 100 pages plain text document produced from online available texts containing the terms ‘digital transformation’ and ‘health services’ in March 2018.

This article is based on the report ‘Assessing the impact of digital transformation of health services’ of the Expert Panel on Effective Ways of Investing in Health (EXPH). Large parts of this article were directly taken from that report. For readability, this is indicated here, without referencing or quotation marks throughout the main text.

The complexity and width of the topic make addressing the impact of the digital transformation a challenging one. Confusion about terminology and concepts, sometimes adds to this challenge. Hence, before turning our attention to this impact and how to evaluate it, it is useful to define a number of key concepts.

Digitalization refers to the use of digital technologies in the context of the production and delivery of a product or service. Such digital technologies allow health care services to be organized, produced and delivered in new ways. Digitalization is therefore less of a ‘technical’ process (like digitization), it is also an organizational and cultural process.

Digitalization, ranging from the use of computers and electronic health dossiers to home monitoring of patients, electronic medical devices, and the application of computer aided visualization and decision support systems, has affected and is expected to affect many aspects of health care systems in terms of structure, culture, professions, treatments and outcomes. This ‘digital transformation’ indicates that health care services and systems are in a transition in which more health services and processes will be digitalized. The digital transformation encompasses the instrumented effort to meaningfully introduce new digital information and communication technologies and corresponding new processes into the health care sector. Some of this digitalization is health care specific, another part is a consequence of the broader digitalization trend in society. Both can lead to changes and innovations in health technologies and health care delivery processes, and thus impact health, health care and health systems. The digital transformation in some of its aspects therefore represents a fundamental change in the mode and culture of care delivery of organizations.

**Figure 2:**
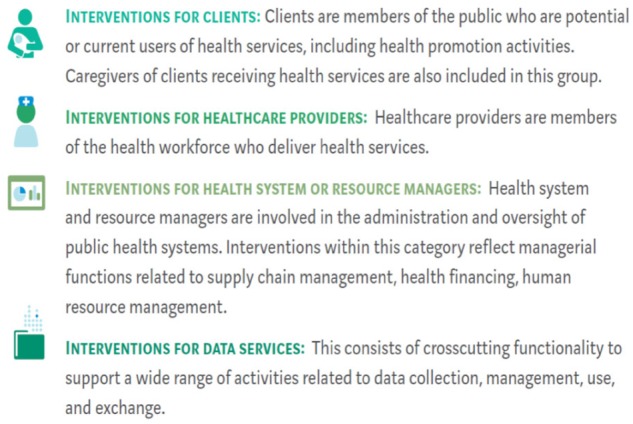
WHO classification digital health services. *Source:* WHO^2^

Recently, WHO provided a classification of digital services, by dividing them into four categories as shown in figure 2, based on main users of the services.

Although much of the digitalization process has yet to take place, it is expected that the impact of digitalization on health, health care delivery and health systems can and will be profound. It will likely (further) affect the different phases of health care delivery, including health promotion, prevention, primary care, specialized care and long term/social care and self-care.

Mobile health services (mHealth) are an example of digital health services that already impact the process of health care delivery. Although more evidence is required, and mHealth in practice takes many forms, there is evidence that it can have a positive impact in certain situations, including asthma treatment and smoking cessation, also in low- and middle-income countries.^3^^,^^4^

The World Economic Forum (WEF) has recently indicated their expectations regarding the profound impact digitalization in health care will have.^5^ It for instance expects that: ‘The healthcare system of the future will look very different, with a crucial change being the move to “consumer-centric” healthcare, allowing citizens to have much more responsibility for managing their healthcare and that of their families’. Such shifts relate to patient empowerment, self-management, shared decision making and also goal orientation of future health care. The directions and diversity of developments relating to digital health services have also been highlighted by the WEF, including aspects like continuous monitoring, connected homes, intelligent treatments and virtual care teams. Note that more specific forecasting is difficult, certainly when it comes to the expected costs and benefits of new technologies.

Digital Health will transform the business models of the Pharmaceutical industry.

Although many companies have not yet formulated a concise Digital Health strategy, industry executives expect that by 2020, Digital Health will enable Pharmaceutical companies to activate new business segments as well as to significantly improve their competitive advantage.

As the WEF emphasises, these developments are likely to entail important shifts from diagnosis and treat, to prevention and management. Moreover, the location of health care delivery may well shift from hospitals and other treatment centres to home. Such shifts, as expected by the WEF, would of course imply fundamental changes in the way health systems are organized and financed, the type of health professionals needed, the role of those professionals and of patients, as well as the health services provided and the process of delivery. All such aspects may also be seen as challenges that need to be overcome in order to facilitate the materialization of these expectations regarding the future of care.

Digital health will also transform the business models of the Pharmaceutical and Biomedical industry as it will enable companies to activate new business segments as well as to significantly improve their competitive advantage.

Although many companies have not yet formulated a concise Digital Health strategy, industry executives expect that by 2020, Digital Health will enable Pharmaceutical companies to activate new business segments as well as to significantly improve their competitive advantage.

While such (expected) developments hold the promise of reducing pressure on the workforce, lowering costs and improving patient centeredness and goal-orientation of care, this does not reduce the need for evaluation. Like for other innovations and (new) technologies, such promises may or may not materialize and potential benefits may also be accompanied by unintended and/or negative (side) effects in the short or long term. Hence, the introduction, implementation, use and funding of digital health technologies should be carefully evaluated and monitored. Monitoring and evaluating these technologies appear to be outpaced by the proliferation of digital health technologies,^2^ partly fuelled by the promise they have to improve health, care and health systems. In the context of publicly funded care systems and public decisions, such evaluation and monitoring are necessary and ideally performed in relation to the goals health systems pursue.

## The challenges for European health care systems

European countries typically pursue health systems goals that include high quality, efficiency, equity, affordability and accessibility of health care.

Balancing and optimizing these goals is a continuous process, due to developments both within and outside the health care domain. It typically involves trade-offs between (potentially conflicting) goals, like affordability and quality, requiring normative judgments from relevant decision makers. One of the factors influencing the performance of health care systems in achieving this goal is technological change, including the ongoing process of digitalization of health services. The latter process may have large consequences for the future of health care delivery and health systems. Many countries struggle with the desire to on the one hand stimulate digitalization and the adoption of digital services, in light of their promise to improve health system performance, and, on the other hand, to steer the process of digitalization in the desired direction and evaluate whether it actually improves health care and health system performance. In that context, it needs to be asserted that the benefits of the process of digitalization of health services outweigh the associated costs (in the broadest sense of the word).

Digital technologies and the digital environment offer new opportunities for delivering health care. As such, they have the potential to transform healthcare services in ways that may contribute in several ways to health system goals. The nature and consequences of digital health services can differ substantially from case to case, emphasizing the complexity of evaluating their contribution.

The results and outcomes of digital transformation of health services will importantly depend on the quality of the process and the involved stakeholders. This includes end-users of digital health services (be it professionals or care users), developers of digital health services, producers of health services and governments. The success of digital transformations requires a sound understanding of the two basic interacting components, i.e. ‘the health service’ and ‘the digital’, at all these different levels. The full process of developments, production, funding, implementation and evaluation requires careful consideration.

The innovative solutions that some digital health services represent can, if designed purposefully and implemented in a cost-effective way, provide better health outcomes and contribute to the sustainability of health systems. However, while digital health services can have this effect, they need not have it. Evaluations and monitoring should establish whether this is the case for specific digital health services. The scope of such evaluations and monitoring needs to be set appropriately. This is underlined by the fact that, like other technologies, digitalization in health care normally affects certain goals or certain groups positively, while at the same negatively affecting others.

EU policies have consistently emphasized the importance of digital solutions such as eHealth and have accentuated positive aspects of how digital innovations can improve integration of care through up to date information channels and deliver more targeted, personalized, effective and efficient healthcare, reducing errors and length of hospitalization. However, a balanced view of the effects of digitalization remains needed and not all forms of digitalization may result in improved care and health system performance. Put differently, a health care service is not good (or bad) just because it is digital.

Public expenditure on health and long-term care has been increasing over the last decades in most countries in the world and is expected to rise even further. A substantial part of the increase has been attributed to the introduction and funding of new technologies in health care, including digital ones. In this context, there is a growing need for robust evidence to support arguments that digital health solutions^2^^,^^6^—and the related new organizational models replacing the old—can bring better health outcomes for citizens and contribute to improving the effectiveness, accessibility and resilience of health systems. Given the diverse forms, usages and impacts of digital technologies in health care (ranging from general use of computers to algorithms designed to assist radiotherapists in detecting cancers, from robotic surgery to computer aided decision models, and from mobile device apps helping patients to self-manage their disease to electronic health records), this requires evaluations on different levels.

Systematic assessment and evaluation of the impact of digital health services is therefore needed. To date, such assessments are relatively scarce, especially those addressing the transformative aspects of healthcare delivery on the organizational and operational level.

The literature on the impact, e.g. of telehealth solutions for chronic conditions suggests that telehealth can reduce hospital admissions and mortality for chronic heart failure patients, improve blood pressure control in patients with hypertension, reduce hospital admissions for chronic obstructive pulmonary disease and improve glycaemic control in diabetes.^7–9^ However, the evidence base concerning cost-effectiveness can be less clear as is the generalizability of such results.

A framework for the governance of the digital transformation of health services and its impact is vital to generate the evidence required for decision-making on stimulating, using and/or funding digital health strategies at various levels in the health care system.

This is an objective for governments globally—not just in Europe. The newly established Global Digital Health Partnership,^10^ which comprises 25 country participants and the WHO, has prioritized collaboration on methodologies for evaluation of the benefits of digital transformation in health. Several European countries are involved in this initiative (www.gdhp.org).

## Governance and governments for digital health

### Centralization vs. decentralization

Health policies encouraging enablers for developing and implementing innovations, contributing to the aims of health care systems, are clearly relevant in the context of digital health services.

This emphasizes that the role of governments exceeds that of evaluating specific technologies to see whether or not they should be funded and implemented, but also (and perhaps especially) should focus on creating incentives that steer the (decentralized) development, adoption and use of technologies that contribute to health system goals.

Actually, in centralized decision making, the (potential) alignment with public goals of the health care sector can be directly ensured.

It needs noting that centralized decision making on reimbursement does not automatically guarantee the adoption, implementation or use of an innovation. Possibilities, willingness and incentives at lower levels in the system need to facilitate this. Centralization of decisions can be demanding in terms of the information required to take a system-wide decision. Moreover, system-wide decisions in some instances may be more difficult to undo, in case they are judged as having been ‘wrong’ ex-post. ‘Wrong’ decisions have also been seen to undermine the credibility of policy decisions and loss of trust in HC digitalization as seen in the NHS cyber attack by the WannaCry virus in May 2017, subsequently slowing or hindering beneficial digitalization.

Decentralized decision making may involve other goals and incentives, both from public and private parties, than overall health system goals. With decentralized decision making, we refer to those decisions that result in the adoption, implementation and use of digital health services without a formal decision to do so by a public entity on a regional or national level. For example, the decision of an individual hospital to implement a specific electronic health dossier is seen as a decentralized decision. Each hospital makes own choices, some of which may be better than others. Even if they are equally good, the fact that different hospitals use different systems may lead to problems of coordination and interoperability. It is acknowledged that decentralized development, adoption and implementation of digital health services can have both advantages (competition, creativity, several pilots, etc.) and disadvantages (unnecessary experimenting, suboptimal outcomes on system level, etc.). Governments may therefore need to take a role in this. Also, if decentralized decisions are less aligned with the overall health system goals, this may require additional government intervention to guide the digital transformation in desirable directions. Monitoring and policies to direct developments in desired directions are crucial in that context. European countries could benefit from developing, implementing and systematically using such evaluation and monitoring systems.

### Multidisciplinary and multi-stakeholders collaboration

The WHO guide emphasis the different stages of the development of a digital health service, the need to involve different stakeholders in the development, monitoring, evaluation and implementation phases. It also highlights how these phases and processes are connected and provides practical guidance on methods and reporting.

The authors highlight that at ‘the core of every digital health system or intervention is a value proposition—a statement describing the benefits to end-users, with an implicit comparator, which can be a non-digital intervention or an alternative digital product. […] Value propositions describe (i) which end-user needs are met by the digital health system and how, (ii) why the digital health system is innovative and (iii) why the digital health system is superior to the standard of care or status quo. […] Claims about the digital health intervention are based on assumptions about end-user needs and/or the effectiveness of the digital health system’.^2^

The claims could include aspects like efficacy, effectiveness or cost-effectiveness and can help to design evaluations and monitoring procedures, because they define the key parameters that are expected to be affected by the new intervention. A point of attention is the possibility of ignoring important unintended consequences of an intervention if the evaluation focuses on the intended and claimed impacts. Hence, a core set of parameters should be included in any evaluation, covering the most important and common impacts. This also increases the comparability of studies.

Multidisciplinary approaches in evaluating health technologies appear to be especially relevant in the context of digital health services. Legal issues, in relation to privacy, information exchange, cross-border care delivery, may require specific attention. Sufficient knowledge on the technical aspects, including issues like scalability, stability and interoperability is also important in the assessment. This can add complexity to evaluations as well. Two digital health services may be equally good, but if suffering from issues of compatibility, using both would still be suboptimal.

Cultural aspects, including issues regarding the acceptability of a technology for patients or professionals (as well as variation in such acceptability) can also be influential.^11^ This is ideally tackled already in the development phase of new digital health services, to ensure an adequate acceptability, uptake and implementation of the technology. Methods for assessing the impact of digital transformation must be also fit for future use. Apart from addressing the existing care services, they must be able to accommodate future paradigm shifts in the goals of health services. A paradigm-shift from ‘disease-oriented’ towards ‘goal-oriented care’ is for instance needed. The goal-oriented care encourages each individual to achieve the highest possible level of health as defined by that individual.^12^ Another paradigm shift resulting from digitalization is oriented at future proactive, predictive, prospective, preventive, participative, personalized health services enabled by improved data use.^13^

### A public health approach

The need to not only consider average progress, but also the distribution of health outcomes, health care use and financial burden in the population should be also emphasized. This is relevant in a general sense, but especially in the context of digital health services which may require specific skills or resources to operate. Although some digital health services (e.g. those strengthening prevention and health promotion) may have the potential of reducing health inequities, others may result in further widening the gap in health achievements between different societal groups. As technical and literacy skills vary greatly between socio-economic and socio-demographic groups, the use of digital health services such as mobile and eHealth technologies could indeed impact negatively and increase social and health inequities.^14^ Issues such as online accessibility, affordability, inadequate digital education and lack of digital literacy constitute real barriers to realizing the potential of digital health interventions for many communities. Reviews of the literature in this area highlight that the ‘digital divide’ encompasses a number of dimensions including; unequal access to digital technologies (internet, mobile phones, etc.); variations in use due to a lack of sufficient knowledge and confidence on how to use the technology adequately; the health information or digital services provided may not be comprehensible or useful for disadvantaged populations.^15^^,^^16^ The empirical studies show that while individuals of higher socio-economic status are the first to adopt and benefit most from the introduction of innovative technologies in health, thereby creating and widening existing inequities, the digital divide tends to affect the same individuals and population groups who are at risk of social and health inequities (low income, low education, low literacy, ethnic minority groups, elderly people, socially marginalized and underserved groups, etc.). It is, therefore, critically important that evaluation studies assess the extent to which digital health technologies may produce, reduce or exacerbate inequities in health.

### Empowering patients and health professionals

Digital health services have the potential to strengthen patient empowerment and provide a more equal basis for shared decision making. Such aspects are valuable assets of a health system.

Involvement of patients in the development and implementation of new digital health technologies in this context is important, in order to optimize their form and impact, as well as to maximize the patient value and acceptance. Governments are starting to implement digital programmes that provide unprecedented citizen control over access to clinical information—designed to empower all in the care pathway to provide higher quality more responsive health and wellbeing outcomes. In February 2019, Australia became the first country in the world to introduce an online My Health Record for all citizens—on an opt out basis (90% of the population is participating). Almost all public health service providers are connected and upload data—access by clinicians is at the discretion of the citizen, who can set record access controls and has a real-time audit history of provider access.

It is important to explicitly note that when new technologies allow more self-care and self-management by patients and health care users, this does not reduce the responsibility of the health care system for these individuals.^17^

Many digital health technologies strongly depend on the uptake and appropriate use by health care professionals. This may lead to new health care professions, as well as to existing health care professionals acquiring new skills and competencies to work with new digital health services. This implies that adequate education and training needs to be in place to enable this. Co-creation in developing new digital health services can be useful to increase acceptability and user friendliness, also in practice. Professionals’ experiences with using the technologies are also crucial to monitor and consider in any evaluation. Some systems may be time consuming to (learn how to) operate, placing additional rather than less strain on often already burdened professionals (in the short or even longer run). Some technologies may also be more or less acceptable (in different ways) for professionals and patients, which is a clear prerequisite for successful implementation and regular use.

Digital health technologies may also change the content and type of professions needed. For instance, when virtual coaches replace human counselling, the responses of the virtual coaches need to be coded. In some cases, digital solutions may replace human labour. This is not desirable or undesirable in itself. Freeing for instance nurses from administrative duties to allow them to spend more time with patients can be good change. This is emphasized by the current and future shortages of staff. Cost reductions brought about by digital health services also do not need to imply lower health care budgets, but simply the possibility to allocate the freed budget elsewhere.

In relation to the previous point, important threats to health care systems include the increase in health care expenditures and the shortage of labour (health care professionals). It is important to highlight that part of the increase of health care expenditures can be related to a difficulty of increasing productivity in the sector, due to the nature of health care (leading to the so-called Baumol effect). Some digital health services may lead to improved productivity and perhaps cost saving, reducing (the growth in) health care expenditures. This is important, also in relation to the potential (and actual) shortage of personnel. If new digital health services can replace some of the functions currently performed by health care professionals, this may relieve some of the pressures due to shortage of personnel. Of course, quality of care needs to be fully considered in this context as well.

## Conclusions

The digital transformation is ongoing, in some cases at a rapid speed. When having to evaluate digital health services or purchase or procure them, but also when monitoring their impact, and preparing (new generations of) health care professionals for the digital transformation, a good knowledge of these technologies also within governments is required. Investment in such knowledge, also in the public domain, is required.

Looking for a method to organize the governance work, the TAPIC framework is one of the most robust in research terms and one of the simplest to use. Drawing on an extensive literature review, the authors of this framework^18^ capture five categories of governance; transparency, accountability, participation, integrity and capacity. These five categories together provide a useful map of the issues that any public body will want to think about when considering their governance arrangements.

For large national digitalization projects, elements like optimal timing, risk sharing, procurement conditions, etc. may also be relevant, which requires sufficient knowledge to act as a well-informed counterpart in negotiations. Especially in such cases, evaluating the new technology is not enough, but negotiations may be required regarding both quality and price. Both aspects are not a given, but part of a purchasing process.

Digital evolution/horizon scanning may offer governments and decision-making bodies with knowledge about the type of products that will come unto the market in the future, so that preliminary decisions can be made about which to evaluate and how to evaluate them, as highlighted above.

Many of the benefits of digitalization will not be realized if the use of patient data is restricted only for the small medical team directly involved in current treatment. Rather, the data should be utilized by the different players in the health system to do so. Thus, in the digital health system there seems to be a constant confrontation between the privacy protection and the data utilization interests.

The recent General Data Protection Regulation of European Union (EU 2016/679), ‘GPDR’ tries to create a balance between privacy rights and the development of the digital market.

Finally, health care is not only utilizing digital solutions but is also becoming dependent on them. This makes the health system susceptible to new kinds of threats. Cybersecurity plays a very important role in ensuring the undisturbed and safe functioning of health care facilities and services.^19^ Electronic health records and other core systems are protected with firewalls and user recognition systems. However, they might still be hacked. Unauthorized users may steal sensitive data or block the utilization of patient records. Use of own mobile devices by health care personnel, remote patient access to health records, wide utilization of applications and electronic devices all increase cybersecurity threats and require specialized expertise to ensure appropriate data protection. Adequate attention to these aspects, that may be central to some digital health services and more complementary to others, is indispensable.
